# Osteoprotegerin secreted by inflammatory and invasive breast cancer cells induces aneuploidy, cell proliferation and angiogenesis

**DOI:** 10.1186/s12885-015-1837-1

**Published:** 2015-11-25

**Authors:** Sudeshna Goswami, Neelam Sharma-Walia

**Affiliations:** Department of Microbiology and Immunology, H. M. Bligh Cancer Research Laboratories, Chicago Medical School, Rosalind Franklin University of Medicine and Science, 3333 Green Bay Road, North Chicago, IL 60064 USA

**Keywords:** Osteoprotegerin, Inflammatory breast cancer, Invasive breast cancer, Tumor microenvironment, Aurora-A kinase, Aneuploidy

## Abstract

**Background:**

Osteoprotegerin (OPG) is a glycoprotein that has multifaceted role and is associated with several cancer malignancies like that of bladder carcinoma, gastric carcinoma, prostate cancer, multiple myeloma and breast cancer. Also OPG has been associated with several organ pathologies. The widespread expression of OPG suggests that OPG may have multiple biological activities that are yet to be explored.

**Methods:**

The anchorage-independent sphere cultures of the adherent cells were instrumental in our study as it provided a deeper insight into the complexity of a 3D tumor. Cytokine profiling was performed for OPG’s detection in the microenvironment. ELISA and western blotting were performed to quantify the OPG secretion and measure the protein levels respectively. OPG expression was detected in human breast cancer tissue samples by IHC. To decipher OPG’s role in tumor aggressiveness both recombinant human OPG as well as OPG rich and depleted breast cancer cell conditioned media were tested. Western blotting and MTT assay were performed to detect changes in signaling pathways and proliferation that were induced in presence of OPG. Onset of aneuploidy, in presence of OPG, was measured by cell cycle analysis and western blotting. Finally, human Breast Cancer qBiomarker Copy Number PCR Array was used to detect how OPG remarkably induced gene copy numbers for oncogenic pathway regulators.

**Results:**

SUM149PT and SUM1315M02 cells secrete high levels of the cytokine OPG compared to primary human mammary epithelial cells (HMEC). High expression of OPG was also detected in human breast cancer tissue samples compared to the uninvolved tissue from the same patient. OPG induced proliferation of control HMEC spheres and triggered the onset of aneuploidy in HMEC sphere cultures. OPG induced the expression of aneuploidy related kinases Aurora-A Kinase (IAK-1), Bub1 and BubR1 probably through the receptor activator of nuclear factor kappa-B ligand (RANKL) and syndecan-1 receptors via the Erk, AKT and GSK3(3 signaling pathway. Gene copy numbers for oncogenic pathway regulators such AKT1, Aurora-A Kinase (AURKA or IAK-1), epidermal growth factor receptor (EGFR) and MYC with a reduction in the copy numbers of cyclin dependent kinase inhibitor 2A (CDKN2A), PTEN and DNA topoisomerase 2 alpha (TOP2A) were induced in presence of OPG.

**Conclusions:**

These results highlight the role of OPG in reprogramming normal mammary epithelial cells to a tumorigenic state and suggest promising avenues for treating inflammatory breast cancer as well as highly invasive breast cancer with new therapeutic targets.

**Electronic supplementary material:**

The online version of this article (doi:10.1186/s12885-015-1837-1) contains supplementary material, which is available to authorized users.

## Background

Breast cancer, the leading cause of death among women with an onset frequency of one in eight, is the most common type of cancer among women. However, despite the advancement in therapy, the mortality rate in breast cancer patients still remains high. The proactive, complex and dynamic tumor microenvironment of breast cancer adds to the grim scenario of the disease by accumulating inflammatory and angiogenic growth factors and creating a niche for the growth and metastasis of breast cancer cells.

The invasive carcinomas in humans can be highly metastasizing, less invasive or localized which indicate the dynamic and progressive nature of breast cancer. Studying the pathobiology of human breast cancer is challenging due to the inherent complexity. To investigate the pathobiology of human breast cancer successfully, it is necessary to maintain and recreate the characteristic three-dimensional (3D) architecture of the tissue in culture since conventional 2D monolayer has many limitations. 3D cultures more closely resemble the *in vivo* situation with regard to cell shape and its microenvironment [1]. It is well established that the development and progression of a tumor toward the malignant phenotype is highly dependent on interactions between tumor cells and its microenvironment. The tumor microenvironment is made up of secreted growth and angiogenic factors, inflammatory cytokines, adhesion molecules, and circulating tumor cells. Tumor microenvironment promotes angiogenesis, cell migration, metastasis, and drives tumor progression to invasive carcinomas [2]. Therefore, in the current study we performed cytokine profiling of breast cancer and healthy mammary cell conditioned media representing their microenvironment. We observed high levels of osteoprotegerin (OPG) secretion from the primary inflammatory ductal carcinoma SUM149PT cells and highly invasive ductal breast carcinoma SUM1315MO2 cells when compared to primary human mammary epithelial cells (HMEC).

OPG, also known as osteoclastogenesis inhibitory factor or tumor necrosis factor receptor superfamily member 11B (TNFRSF11B), is expressed in many tissues such as heart, kidney, liver, spleen, and bone marrow [3]. Besides being an important player in bone metabolism, OPG is a key regulator in vascular disease, prostate cancer, multiple myeloma, breast cancer, bladder carcinoma, and gastric carcinoma [4]. There are multiple evidences suggesting OPG’s association to malignancy [4, 5]. OPG is a multifaceted molecule playing various functional role involved in cancer sustenance and progression such as tumor cell survival [4, 5] resistance to TRAIL induced apoptosis [6], angiogenesis and regulation of cellular phenotype [7]. In this study, we aimed to examine the unexplored role(s) of OPG in aggressive breast cancer progression. We examined whether OPG rich secretions from aggressive breast cancer cells influence healthy HMECs and drive them towards tumorigenesis.

Our studies demonstrate that OPG induces proliferation, angiogenesis, aneuploidy and survival through manipulation of various survival and aneuploidy related kinases in HMEC spheres. Furthermore, OPG upregulated the expression of the cancer initiating cell marker CD24, in HMEC spheres. The biological significance of OPG was confirmed using recombinant human OPG, OPG rich or OPG depleted conditioned medium from breast cancer cells. Overall, our study reveals OPG as a potential therapeutic target for inflammation and invasion related aggressive breast cancer.

## Results

### Breast cancer spheres are phenotypically different from the spheres cultured from normal mammary epithelial cells

**Fig. 1:**
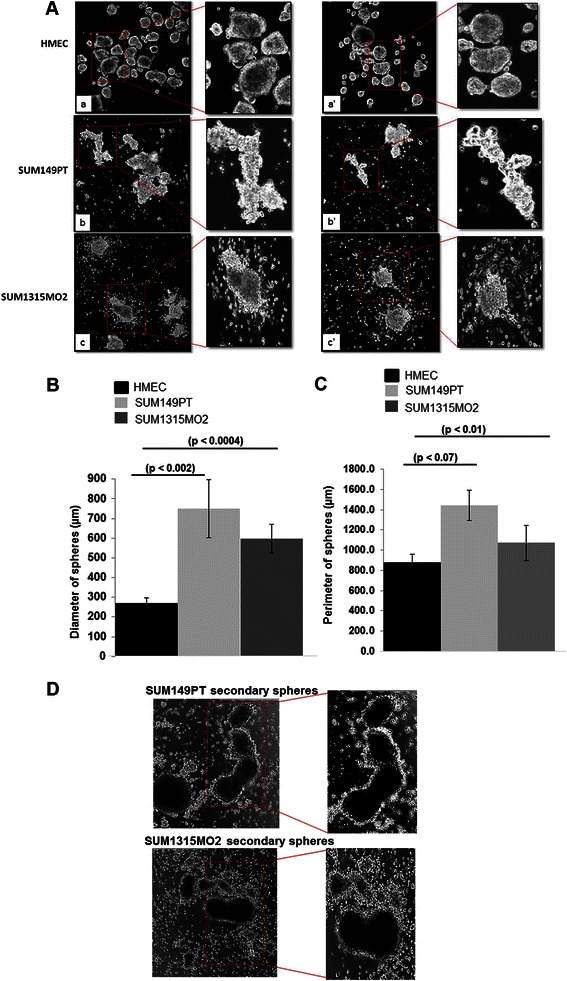
Phenotypic differences between breast cancer spheres and control HMEC spheres. **a** Confocal of HMEC (*a, a’*), SUM149PT (*b, b’*) and SUM1315MO2 (*c, c’*) spheres. Cells were seeded in a 6-well ultra-low attachment plate, grown for 8 days, and then confocal imaged at 10X magnification. Results shown are tiled shots of two different fields and the assay was performed in triplicate with n = 3. The (**b**) diameter and (**c**) perimeter of spheres was measured using the FV10-ASW3.0 software, and statistical significance was calculated with respect to HMEC. **d** Confocal images of secondary sphere cultures from SUM149PT (*a*) and SUM1315MO2 (*b*) with n = 3

Sphere cultures of HMEC, SUM149PT and SUM1315MO2 cells were imaged (Fig. 1a). The control HMEC spheres were heterogeneous in size and comprised of both big and small sized spheres with a smooth periphery (Fig. 1a. a and a’). In contrast, SUM149PT and SUM1315MO2 spheres were bigger in size with a different morphology and highly irregular periphery (Fig. 1a. b, b’, c, and c’). Breast cancer sphere images clearly demonstrate the adherence of multiple spheres forming bigger size spheres. The milieu of the breast cancer spheres seems highly active with new cells colonizing around and homing onto the bigger spheres when compared to HMEC spheres. Using the FV10-ASW3.0 software, we quantified the length and perimeter of the spheres. When compared to the diameter (225 μm) and the perimeter (800 μm) of the HMEC spheres, the breast cancer spheres were significantly bigger in diameter (600–700 μm) as well as in their perimeters (1100–1500 μm) (Fig. 1b and c).

To assess that the morphological changes are due to the microenvironmental factors, the primary spheres of SUM149PT and SUM1315MO2 were trypsinized and reseeded for the growth of secondary spheres and were observed after 10 days (Fig. 1d). Confocal microscopy was performed, which highlighted the adherence of multiple spheres forming bigger size spheres as observed previously (Fig. 1a). Also there was a change in morphology of spheres which can be attributed to the microenvironment that is enriched with various cytokines affecting the molecular and cellular genotype and phenotype.

### Breast cancer adherent cells and spheres have unique a cytokine rich tumor microenvironment

**Fig. 2:**
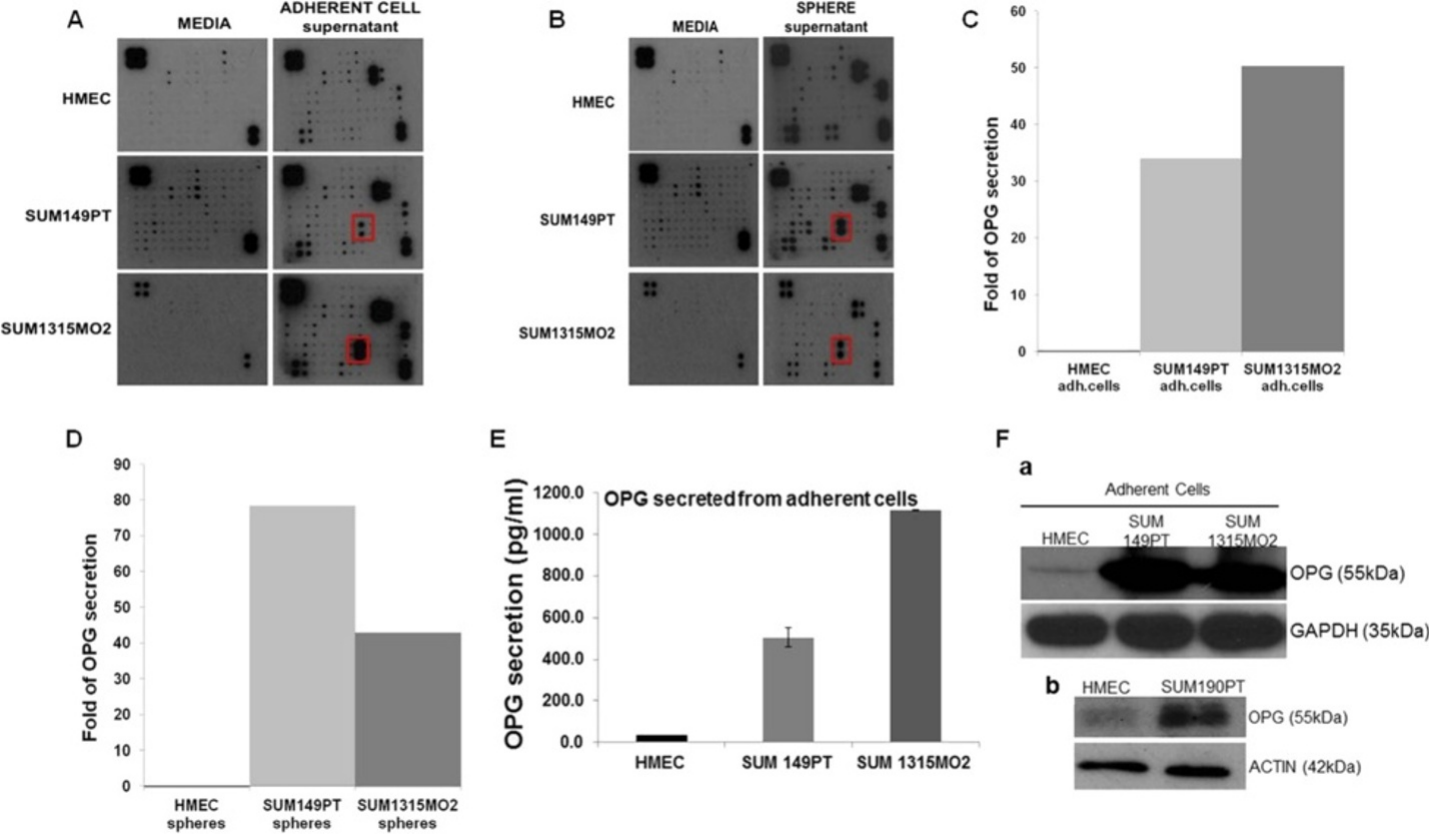
Cytokine array analysis of the conditioned media from (**a**) adherent cells and (**b**) sphere cultures of HMEC, SUM149PT and SUM1315MO2 cells. Red box in the panel indicates the spot on the array corresponding to OPG. **c** and **d** Semiquantitiative densitometric analysis of the OPG in the supernatants obtained from adherent cells and sphere cultures of HMEC, SUM149PT and SUM1315MO2. **e** Conditioned media from adherent HMEC, SUM149PT and SUM1315MO2 cells were collected and analyzed by OPG ELISA. **f** Lysates from adherent cells of (*a*) HMEC, SUM149PT and SUM1315MO2 or (*b*) HMEC and SUM190PT were Western blotted for OPG, stripped and immunoblotted for (*a*) GAPDH or (*b*) β-actin used as loading control. A representative blot from three independent experiments is shown

We reasoned that the highly dynamic milieu of breast cancer cells might be due to a unique cytokine composition of their tumor microenvironment. Hence, we performed cytokine profiling of the conditioned media obtained from adherent cells (Fig. 2a) and sphere cultures (Fig. 2b) of HMEC, SUM149PT, and SUM1315MO2 cells. The tumor microenvironment of breast cancer cells and spheres varied in composition of cytokine and chemokines involved in adhesion and angiogenesis when compared to HMEC cells and spheres (Additional file 1: Figure S1). Most importantly, secretion of OPG, growth related oncogene (GRO) and GRO-α was higher in breast cancer cells and spheres (Additional file 1: Figure S1). When compared to HMEC adherent cells, OPG secretion was increased by 34 fold and 50 fold in SUM149PT and SUM1315MO2 cells, respectively (Fig. 2c and d). Strikingly, OPG secretion from SUM149PT and SUM1315MO2 spheres was increased by 78 fold and 43 fold respectively, when compared to HMEC spheres (Fig. 2c and d). A similar trend was observed in inflammatory cytokine and chemokine composition in the secretions of breast cancer cells and spheres. A highlight was the excessive secretion of highly chemotactic interleukin IL-8 in the breast cancer cells and spheres conditioned media in comparison to HMEC (Additional file 1: Figure S1). The secretion of other angiogenic and growth factors like Growth related oncogene (GRO), Growth related oncogene-alpha (GROα), urokinase plaminogen activator receptor (uPAR), Oncostatin M (OSM), soluble tumor necrosis factor-alpha receptor 1 (sTNFRI), Intercellular adhesion molecule 1 (ICAM-1) was increased in the conditioned media of breast cancer cells when compared to control HMEC cells conditioned media (Additional file 1: Figure S1). The inflammatory chemokines and cytokines like Glucocorticoid induced TNFR related protein (GITR), Haemoinfiltrate CC chemokine 4 (HCC-4), Thymus expressed chemokine (TECK), Interleukin 17 (IL-17), Interleukin 6 receptor (IL-6R) and other cytokines like TIMP metallopeptidase inhibitor 1 (TIMP-1), TIMP metallopeptidase inhibitor 2 (TIMP-2), Fas were also getting secreted (Additional file 1: Figure S1). Similar trend was observed in the microenvironment of breast cancer spheres when compared to HMEC spheres (Additional file 1: Figure S1).

Results of cytokine profiling (Additional file 1: Figure S1) clearly indicate the striking differences between the cytokine profile as well as the level of cytokine secretion between adherent cells and sphere cultures. These results demonstrate the probable complexity and difference in 3D sphere biology in comparison to 2D adherent cell biology. Since cytokine profiling is semi-quantitative, we therefore quantified the secretion of OPG by ELISA as described in the methods. Compared to HMEC, 500 and 1100 pg/ml of OPG was secreted in the conditioned media of SUM149PT and SUM1315MO2 cells, respectively (Fig. 2e). Western blot analysis also confirmed that the OPG level was significantly high in the adherent breast cancer cells when compared to HMEC cells (Fig. 2f. a).

To further validate the observation, Western blot analysis was performed in a different inflammatory breast cancer cell line SUM190PT which also revealed the high levels of OPG when compared to the HMEC cells (Fig. 2f. b).

### OPG expression is significantly elevated in the breast cancer tissue

**Fig. 3:**
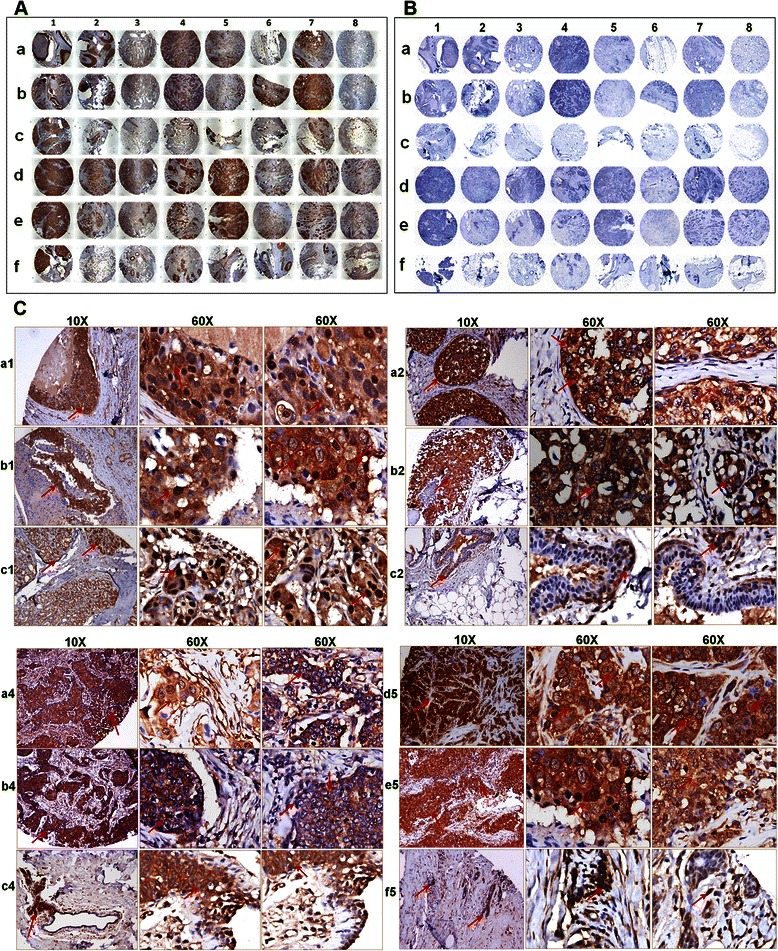
OPG expression in human invasive ductal carcinoma breast tissue samples. **a** 16 breast cancer tissue samples, in duplicates (panel *a*, *b* and panel *d*, *e*) along with their controls (panel *c* and *f*) were analyzed by IHC staining for OPG. Magnification for the panels is 4X. **b** Isotype control staining of the same breast cancer tissue samples. **c** Magnified view (10X and 60X) of OPG staining in selected human breast cancer tissue samples. *Red arrows* indicate OPG staining

To extend our previous *in vitro* observations, we analyzed the breast tissue sections of healthy subjects and invasive ductal carcinoma breast patients for the presence of OPG by immunohistochemical staining using anti-OPG antibody. Most of the abundant OPG expression was detected in invasive ductal carcinoma breast tissue sections (Fig. 3a, panels a, b and d, e) compared to the normal healthy control tissue section (Fig. 3a, panel c and f). Specificity of OPG staining was confirmed by the non-reactivity of isotype control for OPG antibody (Fig. 3b). There was a consistent high expression of OPG in the invasive ductal carcinoma breast tissue samples (Fig. 3c, panels a1, b1, a2, b2, a4, b4, d5 and e5) when compared to the control uninvolved tissue samples (Fig. 3c, panels c2, c4 and f5). However, there was one exception where the control uninvolved tissue samples showed abundant expression of OPG (Fig. 3c, panel c1). We next evaluated the fold OPG expression in all 32 tumor sections by densitometric analysis using ImageJ software. 0–2, 2–4, 4–6 fold induction in OPG expression was observed in 13, 13 and 6 tumor sections, respectively (Additional file 2: Table S1). Collectively these results for the first time show the presence of OPG expression in invasive ductal carcinoma breast tissues.

**Fig. 4:**
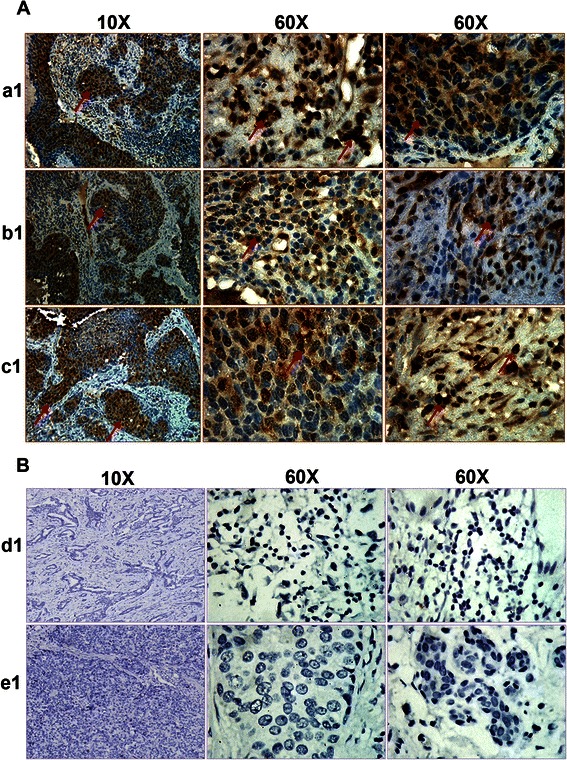
OPG expression in human inflammatory breast cancer tissue samples. **a** Breast cancer tissue samples were analyzed by IHC staining for OPG. Magnification for the panel is 4X and 60X. **b** Isotype control staining of the same breast cancer tissue samples. *Red arrows* indicate OPG staining

Similarly, staining the inflammatory breast cancer patient tissue sample using anti-OPG antibody revealed specific abundant OPG staining (Fig. 4a, panels a1, b1 and c1). The specificity of OPG staining was confirmed by the non-reactivity of isotype control for OPG antibody (Fig. 4b, panels d1 and e1). Taken together the immunohistochemistry results highlight the abundant OPG expression in two different types of breast cancers that is the invasive and inflammatory breast cancer.

### OPG induces in vitro tube formation in primary endothelial cells

**Fig. 5:**
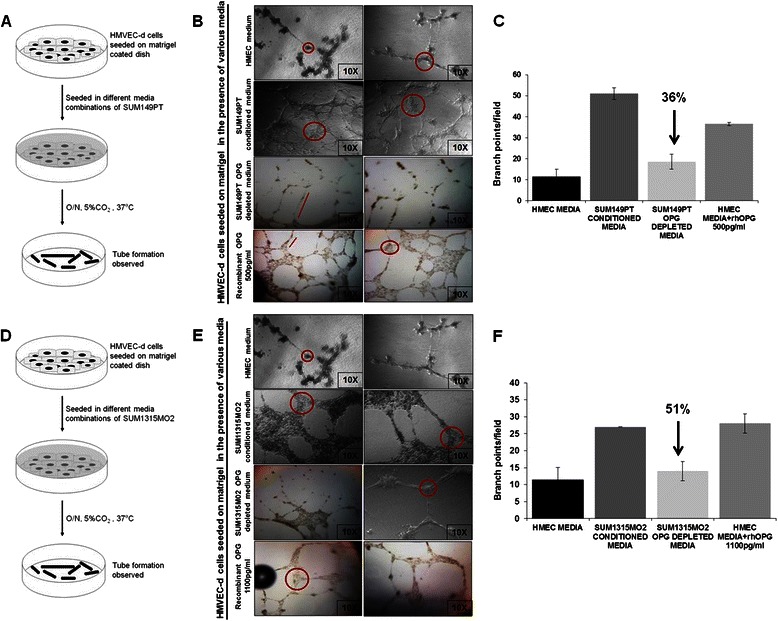
Effect of OPG on HMVEC-d capillary tube formation. **a** and **d** Schematic of the angiogenesis experiment. **b** and **e** Photomicrographs showing HMVEC-d cell tube formation with various supernatants in a matrigel coated 96-well plate. Panels showing angiogenesis in the presence of conditioned media obtained from HMEC, SUM149PT cells, OPG depleted SUM149PT conditioned media, SUM1315MO2 cells, OPG depleted SUM1315MO2 conditioned media and recombinant human OPG media. After 16 h incubation, plates were examined for capillary tube formation under an inverted microscope and photographed at 10X magnification. Circles represent branch points/field (connections among cells), and the lines represent length of the capillary tubes. Each assay was done in triplicate and each experiment was repeated three times. **c** and **f** Quantitative representation of branch points/field in the presence of the different media combinations. Fold increase in the branch points/field was calculated considering branch points/field in presence of control HMEC media as 1 fold

Previous studies have demonstrated that OPG acts as an autocrine and paracrine factor [8, 9]. We hypothesized that the OPG rich tumor microenvironment via its paracrine actions reprograms normal mammary epithelial cells to a tumorigenic state. Therefore, we next studied the effect of OPG on the *‘in vitro sphere cultures’* of HMEC as a working model. We depleted OPG from the conditioned media of SUM149PT and SUM1315MO2 adherent cells by using OPG depleting antibody [10]. 98 and 95 % depletion was observed in SUM149PT and SUM1315MO2 media respectively in comparison to the OPG rich breast cancer conditioned media (Additional file 3: Table S3). Since the tumor microenvironment can facilitate angiogenesis, we evaluated the role of OPG in regulating angiogenesis using an HMVEC-d cell tube formation assay as described in the schematic Fig. 5a and d. A dense network of *in vitro* tubes was observed when conditioned media from SUM149PT (Fig. 5b) and SUM1315MO2 (Fig. 5e) were used. The length, width, and number of nodes were increased in the presence of conditioned media from SUM149PT (Fig. 5b) and SUM1315MO2 cells (Fig. 5e) when compared to the conditioned media from HMEC cells (Fig. 5b). In contrast, the network formed in the presence of OPG-depleted breast cancer cell conditioned media was highly primitive and the tubes were poorly defined (Fig. 5b and e). The length and the width of the tubes as well as the number of nodes significantly decreased in the presence of OPG-depleted breast cancer cell conditioned media (Fig. 5b and e). In the presence of recombinant human OPG, an intricate network of long tubes with multiple node formation was observed (Fig. 5b and e). Quantitatively, conditioned media from SUM149PT and SUM1315MO2 adherent cell conditioned medium induced ~ 5-fold and 2.5 fold, respectively more branch points/field than HMEC medium (Fig. 5c and f). However, OPG-depleted SUM149PT and SUM1315MO2 conditioned media reduced node formation by 36 and 51 %, respectively (Fig. 5c and f). Interestingly, in the presence of 500 pg/ml and 1100 pg/ml of recombinant human OPG ~ 3.7 fold and 2.5 fold more branch points/field formation was induced when compared to HMEC medium (Fig. 5c and f). Taken together, our results suggest that OPG immensely contributes to the angiogenic signature of aggressive breast cancer tumor microenvironment.

### OPG induces proliferation in HMEC spheres

**Fig. 6:**
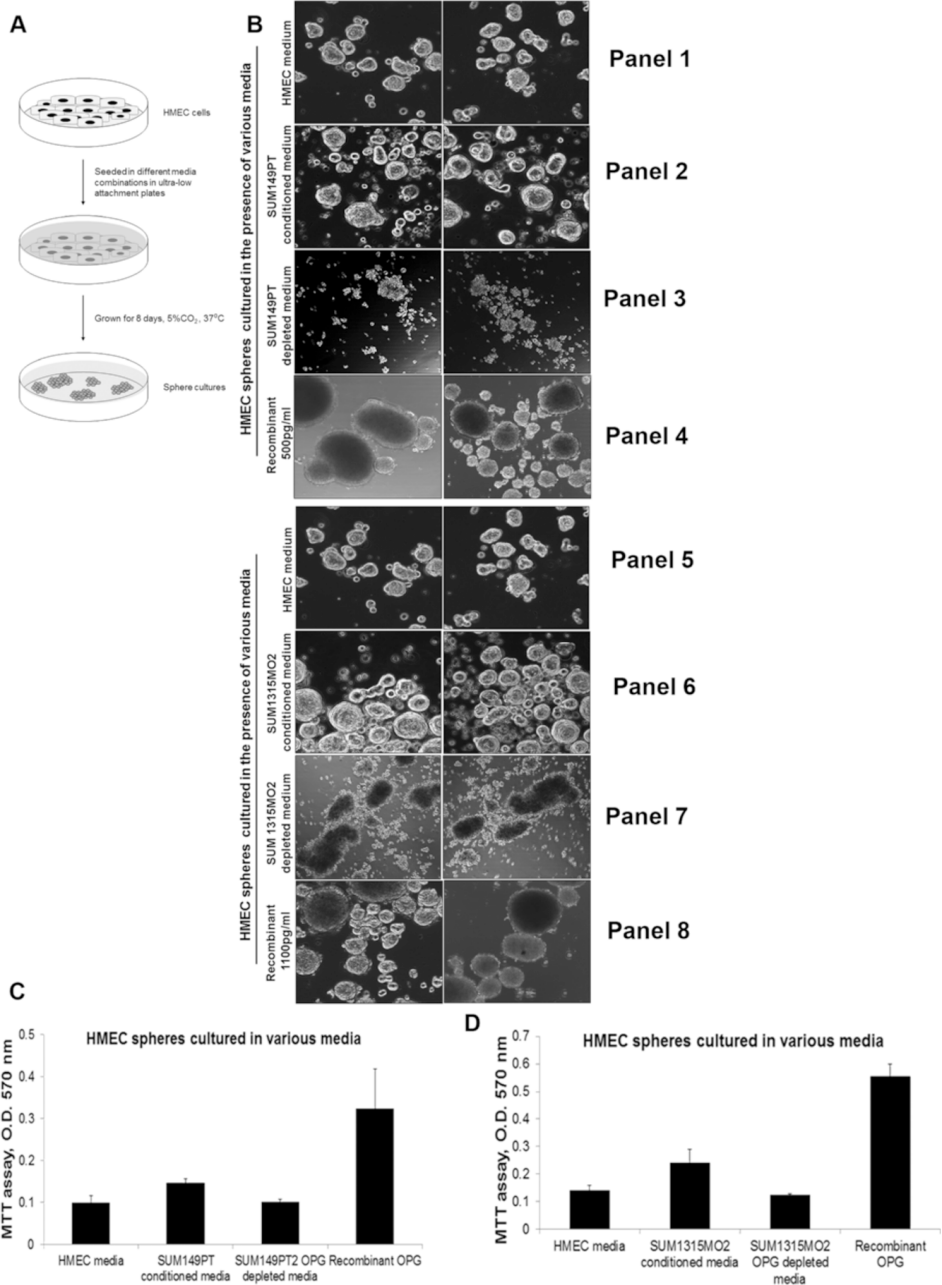
Effect of Osteoprotegerin on the proliferation of HMEC spheres. **a** Schematic of the experiment. **b** Confocal images of control HMEC spheres grown in various media combinations. HMEC cells were seeded in various media in 6 well ultra-low attachment plates as indicated, grown for 8 days, and imaged (magnification, 10X). Results shown are tiled shots of two different fields and the assay was performed in triplicate. **c** and **d** OPG increases the proliferation of HMEC spheres. MTT assay results shown in the panel represent the absorption at 570 nm. HMEC spheres grown in various supernatants for 8 days as described in methods. The values correspond to the mean +/− S.D. of three independent experiments

Since it has been previously shown that OPG acts as an important survival factor for cancer cells [10], we examined the effect of OPG on the proliferation of HMEC spheres (Fig. 6a). Confocal microscopy revealed that proliferation of the HMEC spheres was drastically increased when seeded in the presence of breast cancer cell conditioned media rich in OPG (Fig. 6b, panels 2 and 6). Such spheres were higher in number, bigger in size, and were highly dense when compared to the control HMEC spheres (Fig. 6b, panels 1 and 5). However, this was not the case when OPG-depleted breast cancer conditioned media was used (Fig. 6b, panels 3 and 7). Interestingly, in the presence of recombinant human OPG, the spheres were bigger, denser and more in number (Fig. 6b, panels 4 and 8). Quantification of the proliferation was done by MTT assay and the proliferation index of the HMEC spheres significantly increased in the presence of recombinant human OPG (Fig. 6c and d). These results correlate with the findings using breast cancer cell conditioned media that is rich in OPG and thus suggest that OPG plays a very important role in cell proliferation.

### OPG affects the CSCs population by downregulating the CD24 receptor expression

**Fig. 7:**
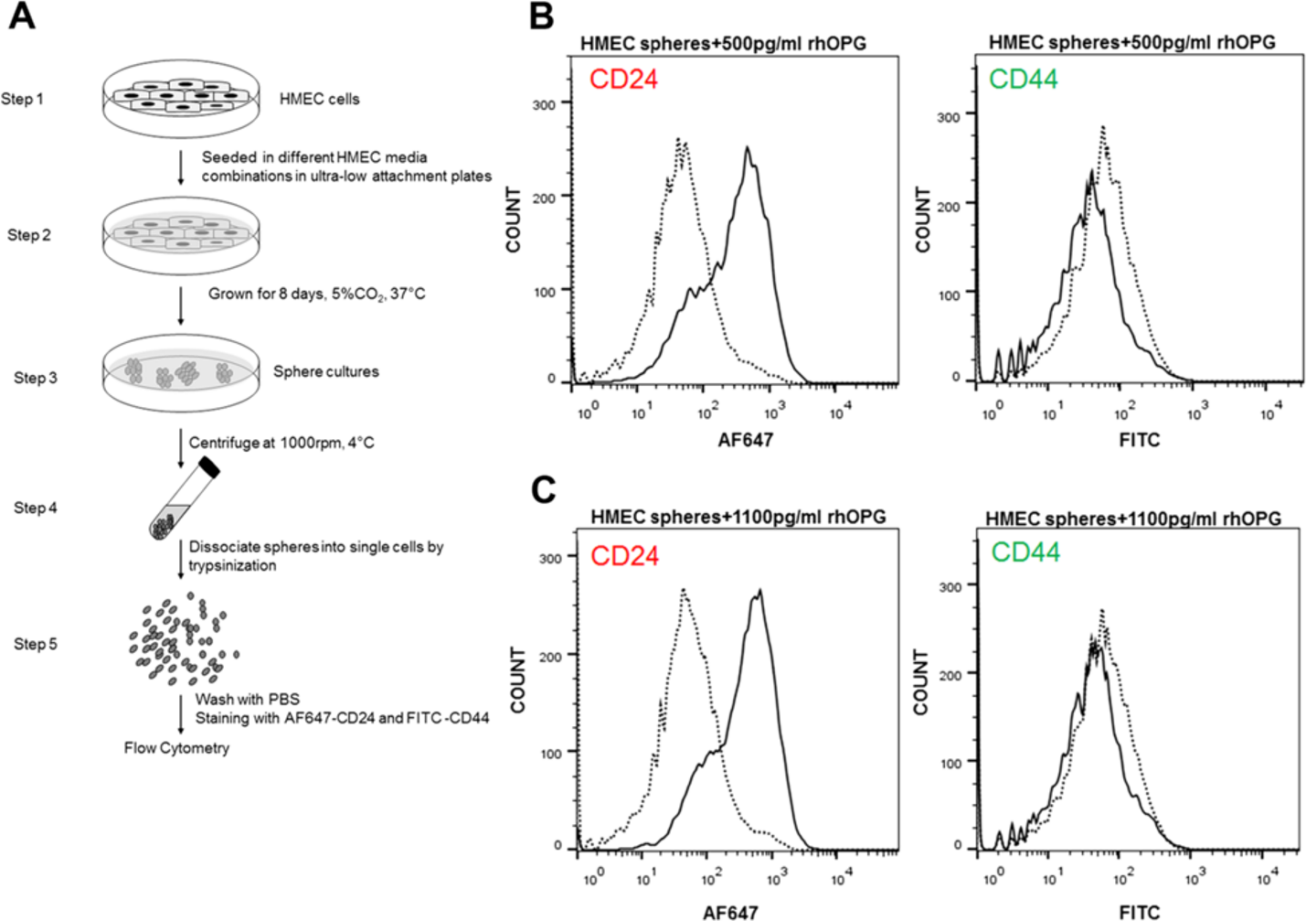
Effect of Osteoprotegerin on the CD44/CD24 CSCs of control HMEC spheres. **a** Schematic of the experiment. **b** and **c** Flow cytometry of HMEC spheres grown in various media combinations. HMEC cells were seeded in various media in 6 well ultra-low attachment plates as indicated, grown for 8 days, trypsinized and stained with CD44 and CD24 antibodies and analyzed by flow cytometry

Cluster of differentiation 44 (CD44) signaling interactions play a key role in various malignancies, supporting tumor cell migration, adhesion, and survival. CD24 is expressed in many cancer types, including renal, ovarian, lung and pancreatic cancers [11–13]. Currently, CD24 expression is a new prognostic marker in breast cancer [12]. ). The CD24 overexpression is linked to worse survival outcomes and increased **aggressiveness of the disease by promoting cell migration and invasion** [14]. Since we observed increased proliferation in the control HMEC spheres in presence of recombinant human OPG, we analyzed if OPG is able to induce any change in the CD44/CD24 profiling of the control HMEC spheres (Fig. 7). HMEC cells were grown into spheres in the presence of various media combinations such as HMEC media and HMEC media supplemented with recombinant human OPG (500 pg/ml or 1100 pg/ml) as shown in the schematic figure (Fig. 7a). Flow cytometry analysis of CD44 and CD24 surface receptor expression revealed upregulation of the CD24 surface receptor expression in presence of 500 pg/ml (Fig. 7b) as well as 1100 pg/ml (Fig. 7c) of rhOPG. However, not much difference was observed in CD44 surface receptor expression (Fig. 7). From our previous experiment we have observed increased proliferation in control HMEC spheres in presence of OPG. Interestingly, CD24 is known to enhance cell migration and proliferation via regulation of signaling molecules like Cten, FAK and ERK1/2 in colorectal cancer. Su et al. (2012) [15] and Weichert et al. (2005) [16] and it’s ablation resulted in decrease in cell proliferation and epithelial to mesenchymal transition via the Src/FAK /ERK signaling via integrin β1 (CD24 regulates cell proliferation and transforming growth factor β‐induced epithelial to mesenchymal transition through modulation of integrin β1 stability). From our previous experiment we have observed increased proliferation in control HMEC spheres in presence of OPG. Thus observing upregulation of CD24 surface expression observed in control HMEC spheres in presence of OPG (Fig. 7b and c) validates the observed increased/sustained proliferation in such spheres as described in Fig. 6c and d.

### OPG induces onset of aneuploidy in normal HMEC spheres

**Fig. 8:**
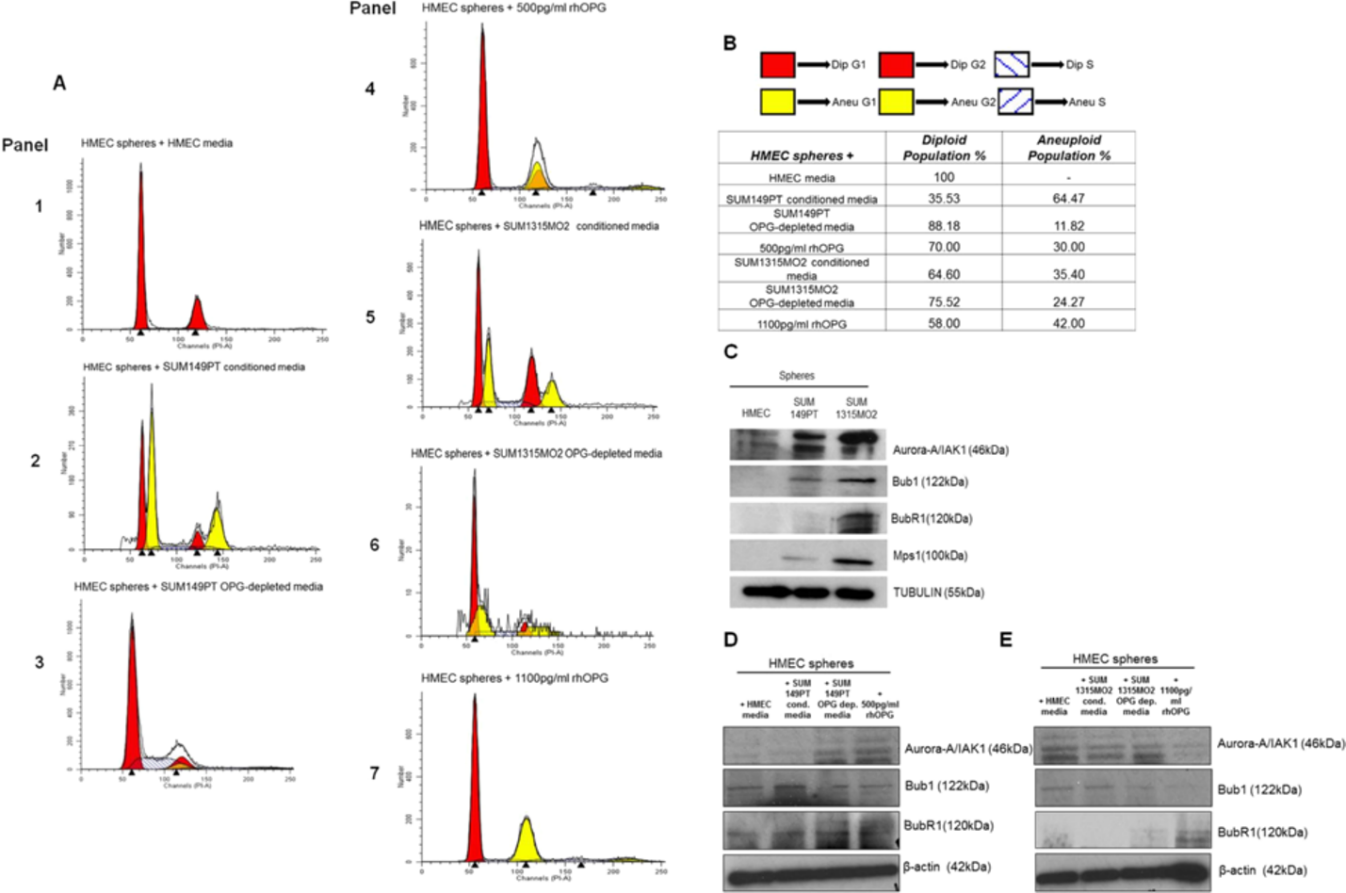
Effect of Osteoprotegerin on HMEC spheres cell cycle and aneuploid kinases. **a** Cell cycle analysis of HMEC spheres by PI staining. HMEC spheres were grown in HMEC media, SUM149PT conditioned media, OPG depleted SUM149PT conditioned media, HMEC media reconstituted with 500 pg/ml recombinant human OPG, SUM1315MO2 conditioned media, OPG depleted SUM1315MO2 conditioned media and HMEC media reconstituted with 1100 pg/ml recombinant human OPG in 6 well ultra-low attachment plates for 8 days. Cell cycle analysis was performed after PI staining of the HMEC spheres. Each assay was done in triplicate and each experiment was repeated three times. **b** Diploid and aneuploid populations of HMEC spheres in the presence of the various media combination as indicated. **c** Western blot analysis of Aurora-A/IAK-1, Bub1, BubR1 and Mps1 in the HMEC, SUM149PT and SUM1315MO2 sphere culture lysates. **d** and **e** Western blot analysis for aneuploidy related kinases. HMEC spheres were grown for 8 days in different media combinations as indicated and total cell lysates were immunoblotted for Aurora-A/IAK-1, Bub1, and BubR1. The levels of aneuploidy kinases were normalized with respect to β-actin used as the loading control

Aneuploidy is a striking hallmark of cancer and studies have highlighted the fact that acquired aneuploidy may be a specific phenomenon in tumor progression [17, 18]. Since we observed increased proliferation in the control HMEC spheres, we analyzed if OPG secreted in the breast cancer cell microenvironment induces aneuploidy. To assess the onset of aneuploidy, HMEC cells were grown into spheres in the presence of various media combinations such as HMEC media, breast cancer cell (SUM149PT and SUM1315MO2) conditioned media, OPG depleted breast cancer cells conditioned media, and HMEC media supplemented with recombinant human OPG (500 pg/ml or 1100 pg/ml). PI staining and cell cycle analysis demonstrated that control HMEC spheres were 100 % diploid (Fig. 8a, panel 1) when grown in HMEC media. Strikingly, when HMEC spheres were grown in SUM149PT or SUM1315MO2 conditioned media, there was an onset of a new population with a decrease in the percentage of diploid population (Fig. 8a, panels 2 and 5). In contrast, the percentage of the aneuploid population decreased in the presence of OPG depleted breast cancer conditioned media (Fig. 8a, panels 3 and 6). Surprisingly, recombinant human OPG (500 pg/ml or 1100 pg/ml) also induced aneuploidy in the control HMEC spheres (Fig. 8a, panels 4 and 7). These results (Fig. 8b) suggested that increased aneuploidy can also be linked to increased cell proliferation and OPG is one of the key factors causing aneuploidy in otherwise normal healthy cells.

### OPG induces aneuploidy related kinases in normal HMEC spheres

To determine factors that may induce aneuploidy, we tested the basal levels of IAK-1/Aurora A, Bub1, BubR1 and Mps1 aneuploidy kinases in HMEC and breast cancer spheres (Fig. 8c). IAK-1/Aurora A, Bub1, BubR1 and Mps1 aneuploid kinase levels were upregulated in breast cancer spheres when compared to HMEC spheres (Fig. 8c). To further verify our results, we evaluated the status of aneuploidy kinases such as IAK-1/Aurora A, Bub1 and BubR1 (Fig. 8d and e) in HMEC spheres grown in different media combinations as indicated. The expression of IAK-1/Aurora A, Bub1, and BubR1 were upregulated in the HMEC spheres grown in OPG rich breast cancer cell conditioned media and recombinant human OPG (Fig. 8d and e). These results complemented the cell cycle results further strengthening the role of OPG in affecting the genomic integrity of normal healthy HMEC cells.

### OPG induces survival and proliferation kinases in HMEC spheres

**Fig. 9:**
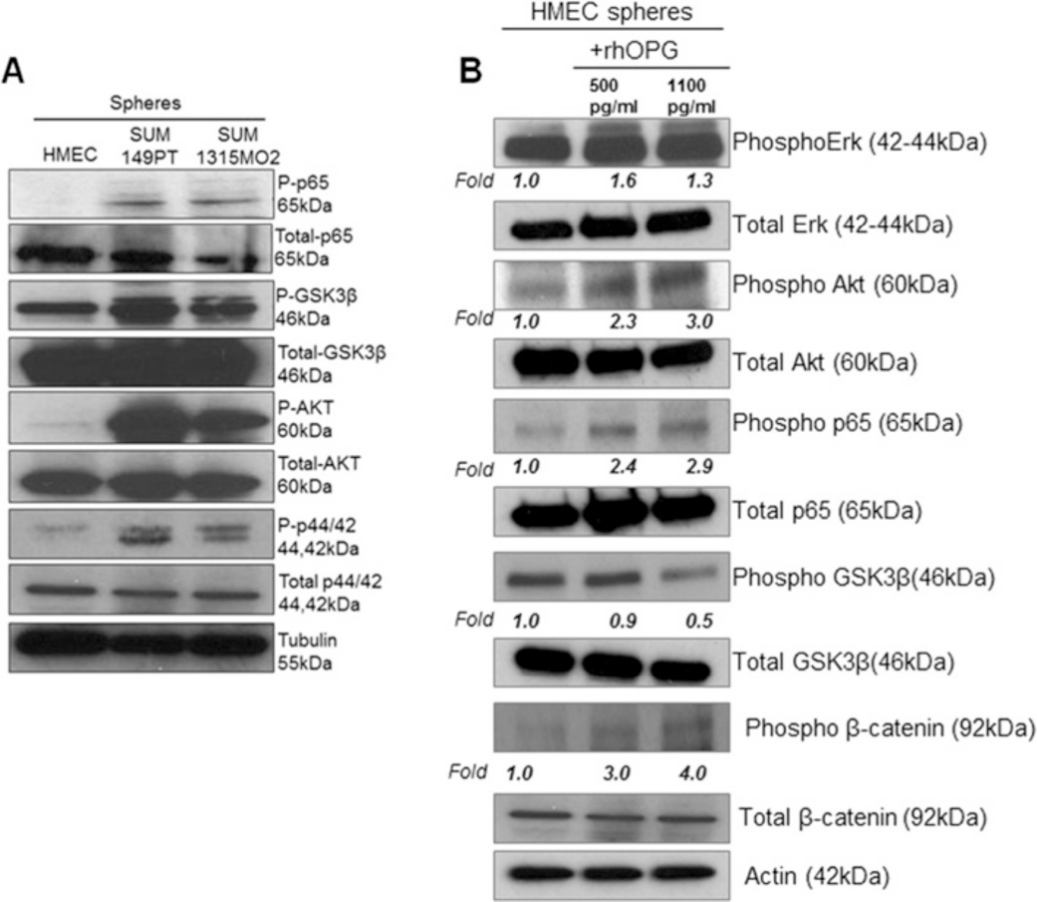
Effect of OPG on survival kinases. **a** Western blot analysis of total cell lysates from HMEC, SUM149PT and SUM1315MO2 sphere culture grown for 8 days. RIPA lysates were immunoblotted for P-AKT, P-p44/42, P-GSK3β and P-p65, and normalized with respect to the levels of total protein levels. **b** RIPA lysates from HMEC spheres grown in different media combinations were immunoblotted for P-p65, P-GSK3β, P-AKT and P-p44/42, and normalized with respect to the total protein levels. The phosphorylation fold has been calculated quantitatively by ImageJ. Actin was used as the loading control

Since increased proliferation could be attributed to the activation of cell survival kinases, we compared the phosphorylated and total levels of survival kinases such as AKT, p44/42, p65 and GSK3β in HMEC and breast cancer spheres (Fig. 9a). Level of P-AKT, P-p44/42, P-p65 and P-GSK3β were higher in SUM149PT and SUM1315MO2 spheres when compared to the HMEC spheres. Due to the increased proliferation and onset of aneuploidy observed in HMEC spheres in presence of recombinant human OPG, we hypothesized that OPG upregulates different survival and proliferation kinases which drive normal cells towards uncontrolled proliferation and survival, an important hallmark of tumorigenesis. To test this hypothesis, in the presence of various media combinations such as HMEC media and HMEC media supplemented with recombinant human OPG (500 pg/ml or 1100 pg/ml) and lysates were prepared. AKT, p44/42, p65, β-catenin and GSK3β phosphorylation levels were increased in the HMEC spheres grown in presence of the HMEC media supplemented with recombinant human OPG (500 pg/ml or 1100 pg/ml) (Fig. 9b). This shows that the phosphorylation of survival and proliferation inducing kinases is increased in control HMEC spheres in the presence of OPG. This finding suggests that OPG upregulated the expression of the survival kinases which probably lead to the increased proliferation of control HMEC spheres.

### Long term culturing of HMEC spheres in OPG rich medium amplified genes relevant to oncogenic pathways

**Fig. 10:**
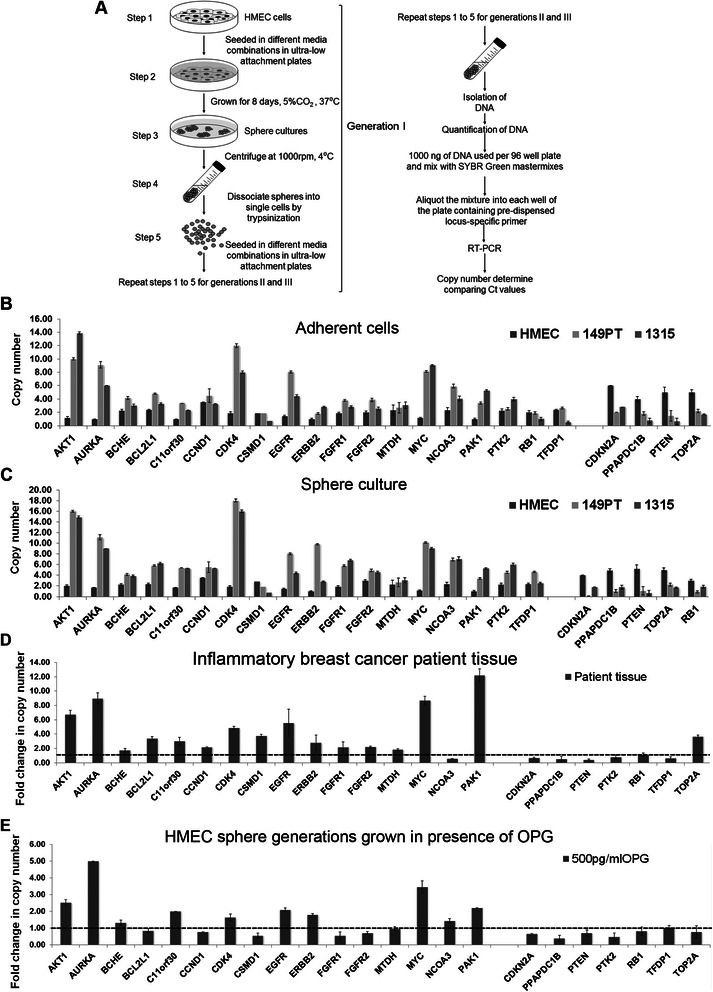
Effect of long term culturing HMEC spheres in OPG rich medium on the copy number of genes relevant to oncogenic pathways. **a** Schematic of culturing three generations of HMEC spheres in presence of rhOPG. **b** Copy number differences among HMEC, SUM149PT, and SUM1315MO2 adherent cells. **c** Copy number differences among HMEC, SUM149PT, and SUM1315MO2 spheres. **d** Comparison of copy number analysis of reference and inflammatory breast cancer tissue DNA. **e** Comparison of copy number analysis of HMEC spheres cultured for three generations in the absence or presence of 500 pg/ml rhOPG

Beside single nucleotide polymorphisms (SNPs), copy number variations (CNVs) are characterized as common genetic variation especially structural variation has been associated with disease susceptibility and onset of cancer [21]. CNVs are defined as DNA segments that are 1 kb or larger in size present at variable copy number in comparison with a reference genome [22]. These variations are common in the human genome [23] and can have dramatic phenotypic consequences as a result of altering gene dosage, disrupting coding sequences or perturbing long-range gene regulation [24]. Changes in the copy number can be positively [25] or negatively [26] correlated with gene expression levels (for example, deletion of a transcriptional repressor could serve to elevate gene expression). To have a baseline of copy number of various genes in various cell types, first we performed the copy number analysis of adherent HMEC, SUM149PT, and SUM1315MO2 cells (Fig. 10b). We used Human Breast Cancer qBiomarker Copy Number PCR Array that profiles the copy number of 23 genes reported to undergo frequent genomic alterations in human breast tumor DNA. The genes on the array encode receptors, receptor tyrosine kinases (epidermal growth factor receptor; EGFR, ERBB2, basic fibroblast growth factor receptor 1; FGFR1, FGFR2), signal transduction pathways (AKT1, phosphatidic acid phosphatase type 2 domain containing 1B; PPAPDC1B, PTEN), phosphatases, transcription factors (metadherin; MTDH, MYC, nuclear receptor coactivator 3; NCOA3, retinoblastoma protein 1; RB1, transcription factor Dp-1; TFDP1), cell cycle (Aurora A kinase, BCL2L1, CCND1, CDK4, cyclin-dependent kinase inhibitor 2A; CDKN2A, RB1), DNA repair (C11orf30; EMSY, topoisomerase DNA II alpha TOP2A), apoptosis (BCL2L1, MTDH), growth factor signaling (MTDH), drug metabolism (butyrylcholinesterase; BCHE), and cell adhesion and cytoskeleton (CSMD1, PAK1, PTK2) that regulate the breast cancer aggression and its biology. Comparison of copy number analysis of HMEC, SUM149PT, SUM1315MO2 adherent cells revealed high copy numbers of AKT1, AURKA, CDK4, EGFR1, PAK1 and MYC in breast cancer cells as compared to HMEC cells (Fig. 10b). Similarly, there was remarkably high gene expression of AKT1, AURKA, CDK4, EGFR1, ERBB2, PAK1 and MYC copy number in the DNA prepared from sphere cultures of SUM149PT and SUM1315MO2 as compared to HMEC (Fig. 10c). We observed downregulation of the copy numbers of CDKN2A, PTEN and TOP2A in SUM149PT and SUM1315MO2 adherent (Fig. 10b) as well as spheres (Fig. 10c) when compared to HMEC. Results in Fig. 10b and c clearly indicate that SUM149PT, SUM1315MO2 cancer cell lines have selective amplification and downregulation of genes regulating signal transduction, protein tyrosine kinases, cell proliferation, and cell cycle regulation events when compared to HMEC cells.

In order to understand these copy number variation in the context of inflammatory breast cancer, we profiled the copy number variation observed in inflammatory breast cancer tissue (Fig. 10d). We observed the upregulation of copy number of AKT1, AURKA, CDK4, EGFR, FGFR1, MYC, and PAK1 and downregulation of few tumor suppressors (PTEN, RB1, and PTEN) and cell cycle regulator CDKN2A (Fig. 10d).

Breast cancer heterogeneity and complexity occurs as a consequence of the dysregulation of numerous oncogenic pathways as well as many non-genetic factors, including tumor-microenvironment stresses including hypoxia, lactic acidosis, glucose deprivation, and cytokine rich microenvironment [27]. Non-genetic factors of tumor microenvironment/paracrine milieu have been shown to integrate and influence the genetic framework of cancer; therefore we asked if continuous insult from OPG rich microenvironment could drive normal mammary epithelial cells (HMEC) towards tumorigenic. DNA copy number analysis of the HMEC spheres cultured for three generations (Fig. 10a) in OPG containing medium selectively amplified the DNA copy numbers of AKT1, AURK1, EGFR, MYC and PAK1 (Fig. 10e). There was appreciable amplification of the DNA copy numbers of CDK4 (Fig. 10e). DNA copy number profiling revealed remarkable reduction in the copy numbers of CDKN2A, PTEN and TOP2A in HMEC spheres cultured in presence of OPG (Fig. 10e). These results indicate that longer exposure of HMEC spheres to OPG rich microenvironment amplifies DNA copy number of tumorigenic genes (AKT1, AURK1, EGFR and MYC) and downregulates tumor suppressive genes (CDKN2A, PTEN and TOP2A).

## Discussion and Conclusions

Breast cancer patients develop aggressive metastases to secondary organs such as bone marrow and bone [28–30]. Components of the tumor microenvironment, including macrophages, myoepithelial and endothelial cells, and several extracellular matrix (ECM) molecules, have been shown to play critical roles in mammary duct morphogenesis. Hence, the secretions from these cells, such as the cytokines in the of tumor microenvironment; are increasingly recognized as a major regulator of carcinogenesis and also a critical target for therapeutics [31]. Our study highlights the importance of OPG in the breast cancer microenvironment and suggests how OPG has the tremendous capacity to drive normal healthy cells towards tumorigenesis.

The anchorage-independent sphere cultures of otherwise adherent cells were instrumental in our study as it provided a deeper insight into the complexity of a 3D tumor (Fig. 1). The differences in morphology and branching of breast cancer spheres indicated a very dynamic microenvironment and highlighted the complexity of the disease. We observed that the secretions from highly invasive breast cancer adherent and sphere cultures were rich in OPG (Additional file 1: Figure S1). Besides OPG, chemokines such as urokinase-type plasminogen activator receptor (uPAR), Oncostatin M (OSM), and GRO-α, which help in matrix-metalloprotease activation, ECM degradation, and facilitate metastasis, were also heavily secreted in the breast cancer microenvironment (Fig. 2). In addition, the increase in OPG secretion might be an indication that OPG, directly or indirectly, is inducing the secretion of many such oncogenic factors thus contributing to the severity of the disease.

OPG is associated with several organ pathologies such as endometriosis [32], periodontal [33], thyroid disease [34] and coronary heart disease [20, 35]. The widespread expression of OPG suggests that OPG may have multiple biological activities that are yet to be explored. Whether an OPG linked survival system operates *in vivo* remains to be established, but the elevated expression of OPG in tumors is reported to be associated with poor prognosis in gastric carcinoma [36]. In our study, strong OPG expression was observed in inflammatory breast cancer tissues and moderate proportion of invasive ductal breast cancer tissues and this was absent from normal breast tissue. This observation supports the proposition that OPG expression might be universally involved in the severity of various kinds of breast cancer development and progression (Figs. 3 and 4).

Previous research has shown that OPG is actively involved in the tumor progression by aiding in angiogenesis [7] and OPG deficient mice exhibited vascular calcification thus highlighting the involvement of OPG in the active and intricate vascular system [37]. Our in vitro studies demonstrate OPG involvement in endothelial tube formation in an *in vitro* model of angiogenesis which is in concordance with previous studies (Fig. 5).

Previous findings revealed OPG’s ability to attenuate TRAIL-induced apoptosis by activating integrin, focal adhesion kinase (FAK), and Akt signaling thus suggesting that OPG production may provide cells with a survival advantage [38]. Similarly, our results showed that OPG induces proliferation and enhances survival of normal human mammary epithelial cells (Fig. 6). Hence it is possible that OPG upregulates the compensatory signaling mechanisms by binding to its signaling receptors thus mediating HMEC proliferation and increased survival.

CD24 is a marker for breast cancer initiating cell (] M. Al-Hajj, M.S. Wicha, A. Benito-Hernandez, S.J. Morrison, M.F. Clarke, Proceedings of the National Academy of Sciences of the United States of America 100 (7) (2003) 3983–3988) and modulating CD24 expression can influence the cell’s proliferating and metastasis capacity (P. Baumann, N. Cremers, F. Kroese, G. Orend, R. Chiquet-Ehrismann, T. Uede, H. Yagita, J.P. Sleeman, Cancer Research 65 (23) (2005) 10783–10793.) Previous studies have confirmed the CD24 enhanced proliferation and survival of cancer cells (] S.C. Smith, G. Oxford, Z. Wu, M.D. Nitz, M. Conaway, H.F. Frierson, G. Hampton, D. Theodorescu, Cancer Research 66 (4) (2006) 1917–1922). Here we show the CD24 upregulation in presence of OPG in control HMEC spheres (Fig. 7) thus supporting the increased proliferation and survival (Figs. 6 and 8) seen in the control HMEC spheres in presence of OPG.

Aneuploidy has been proposed to initiate tumorigenesis and is a remarkably common characteristic of tumor cells [39]. Indeed, aneuploidy is found in precancerous lesions of the cervix [40, 41], head and neck [42], colon [41], oesophagus [43] and bone marrow [44]. Aneuploidy has also been detected in premalignant breast [45] and skin [46] lesions in experimental animals as well. It has recently been confirmed that Aurora-A overexpression potentiates the oncogene activity of HRAS, not by interfering with the ploidy of the cell or the number of centrosomes but by influencing cell growth [47]. Overexpression of other kinases like Bub1, BubR1, Mps1, and Aurora-B has been observed in a large variety of tumors containing polyploid cells with an abnormal number of centrosomes [17]. Our study for the first time highlights the expression of aneuploid markers like IAK-1, Bub1, and BubR1 in the presence of OPG (Fig. 8). Our study is novel as it reveals OPG as one of the important factors in the breast cancer cell tumor microenvironment that can initiate the onset of aneuploidy in normal human mammary epithelial cells (Fig. 8). Furthermore, future investigations concentrating on the identification of the genetic defects that contribute in driving aneuploid kinases as oncogenes will help in targeting the aneuploid kinases for therapeutics.

Previous studies have reported that OPG induced cytoskeletal changes related to proliferation and migration of endothelial cells were associated with activation of Akt, Erk1/2, and Src [48]. In our study, we found that OPG modulated the canonical survival and proliferation signaling pathways in the control HMEC spheres (Fig. 9). OPG activated Akt and GSK3β phosphorylation without notably affecting Erk1/2 activation (Fig. 9).

OPG has been reported to exert its effects via OPG receptors, such as type II membrane forms of RANKL [49, 50], TRAIL [51] and heparin sulfate containing proteoglycans, such as syndecan-1 [19, 52]. Syndecans are also recruiters of growth factors and metalloproteases [53] and their interactions are regulated by phosphorylation induced clustering and shedding of the extracellular domain [54–56]. The overexpression of syndecan-1 in adenocarcinoma cell lines has been shown to stimulate proliferation. The balance between shedding and phosphorylation induced clustering marks the switch to a proliferative and invasive phenotype [54–56]. Besides RANKL, TRAIL and syndecan-1, integrin mediated signaling has also been highlighted for activation of signaling pathways leading to cell migration and proliferation by OPG [48, 57]. Interestingly, immunoprecipitation of breast cancer cell extracts by OPG antibody revealed a major band at a molecular mass of 110 kDa (unpublished results). Mass spectrometry analysis revealed it to be nucleolin protein (unpublished results). Nucleolin is a multifunctional shuttling protein present in nucleus, cytoplasm, and on the surface of some types of cells [58]. Nucleolin is a major constituent of nucleoli in exponentially growing cells [59] and functions in the organization of nucleolar chromatin [60], packaging of pre-rRNA [61], rDNA transcription [62], and ribosome assembly by shuttling between the nucleus and the cytoplasm [63]. Expression of nucleolin on cell surface has been reported in HeLa cells [22], lymphoblastoid T cells [22], breast carcinoma cells [64, 65], lung [66], and laryngeal epithelial cells [67], and hepatocarcinoma cells [68]. Nucleolin has also been reported to be expressed on the surface of endothelial cells in angiogenic blood vessels [65]. Interaction between nucleolin and OPG in the breast cancer cells adds to another layer of complexity how OPG could be manipulating functions at the nuclear levels, and these studies are ongoing in our lab.

Since DNA copy number changes in cancer cells have prognostic impact [69–73], our studies have translational significance and need to be evaluated further using higher resolution methods. Aurora A kinase, EGFR, AKT/PI3K, MYC amplification and TOP2A gene copy number deletion or mutation has been significantly associated with several clinicopathological parameters and poor prognosis in breast cancer patients and are both a prognostic marker for poor outcome [71, 74, 75]. In our study, we found that OPG induced gene copy numbers for oncogenic pathway regulators such Aurora A, EGFR, AKT/PI3K, MYC (Fig. 10c). Aurora A is suggested to be one of the proliferation potency parameters which is an independent prognostic factor for early invasive breast cancer patients, and OPG long term exposure drastically induced the copy number of Aurora A kinase (Fig. 10c) further supporting our results in Fig. 8. Aurora kinases, centrosomal serine/threonine kinases, play an essential role in chromosome segregation, and their amplification and/or overexpression has been associated with centrosome anomalies and chromosomal instability as well as abrogation of DNA damage-induced apoptotic response and spindle assembly checkpoint override in tumor cells, thus Aurora A is also defined as an oncogene [76]. Most importantly, the genes induced by OPG are oncogenic and are similar to the ones observed in inflammatory breast cancer patient tissue.

**Fig. 11:**
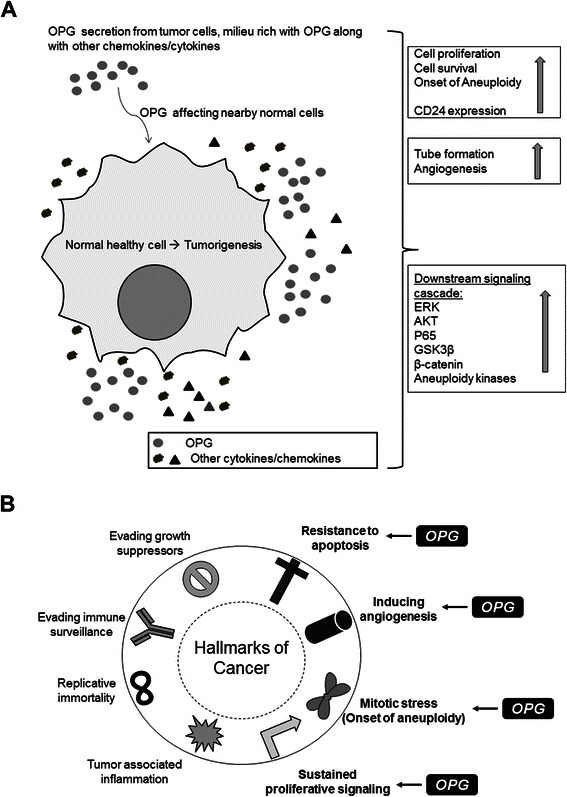
**a** Schematic model depicting that OPG modulates the downstream signaling pathways related to aneuploidy and survival in HMEC cells. **b** OPG promotes tumorigenesis by inducing the different hallmarks of cancer - resistance apoptosis, survival and cell proliferation, angiogenesis and aneuploidy. The paracrine functions of OPG can be mediated via binding to various cell surface receptors on HMEC cells thus driving them towards tumorigenesis

Our study is innovative as it for the first time highlights OPG’s role as an important paracrine factor involved in reprogramming normal healthy cells into tumor cells (Fig. 11) and provides novel information about the possible mechanisms via which OPG activates the downstream signaling pathways thus affecting proliferation, cell cycle and aneuploidy in the normal mammary epithelial cells.

## Methods

### Cells

Primary human mammary epithelial cells (HMEC) (Cell Applications) were cultured in HMEC medium (Cell Applications). Primary inflammatory breast cancer SUM149PT and SUM190PT cells (Asterand), and highly invasive breast cancer SUM1315MO2 cells (Asterand) were grown in F-12 media (Gibco) supplemented with 10 % heat-inactivated fetal bovine serum (HyClone), insulin (Sigma), HEPES (Sigma), EGF (Sigma) for SUM1315MO2 and Hydrocortisone (Sigma) for SUM149PT as per instructions from Asterand. SUM190PT cells were grown in F-12 media (Gibco) supplemented with insulin (Sigma), HEPES (Sigma), Hydrocortisone (Sigma), Apo-Transferrin (Sigma), BSA (Sigma), ethanolamine (Sigma), sodium selenite (Sigma) and 2 % heat-inactivated fetal bovine serum (HyClone). Primary human microvascular dermal endothelial cells (HMVEC-d) (Lonza) were cultured in endothelial basal medium 2 (EBM-2) with growth factors (Lonza). All cells were tested for mycoplasma contamination by the standard Limulus assay (Charles River Endosafe) as per manufacturer’s instructions. All cells were cultured in LPS-free medium.

### Reagents

Antibodies against OPG, Bub1, BubR1 and Mps1 were from Abcam. P-GSK3β, GSK3β, P-p65, P65, AKT, P-AKT, P-p44/42, Erk2 and GAPDH antibodies were from Cell Signaling. Antibodies used against actin and tubulin were from Sigma. The antibody for IAK-1 was purchased from BD Biosciences. Recombinant human OPG from Abcam was dissolved in sterile PBS (pH7.4). For depletion of OPG from conditioned media, the anti-OPG antibody was from R&D Systems, Inc. Antibodies against CD44 and CD24 were purchased from BD Biosciences.

### In vitro sphere culture

Spheres referred as to multicellular tumor spheroids were introduced to in vitro cell culture systems in the early 1970s [77]. Cells were plated at a density of 10^6^ cells/well in a 6-well (Corning) or 10^4^ cells/well in 24-well (Corning) ultra-low attachment plates, grown for 7 days at 37 °C in a humidified atmosphere of 95 % air and 5 % CO_2_ to induce sphere formation. Spheres were collected by centrifugation after 8 days.

### Immunohistochemistry (IHC)

Sections from breast tissue samples of healthy subjects and patients were obtained from Biochain Institute, Inc. (breast tumor tissue array Z7020007). The tumor diagnosis and tumor grading (stages I-III) for the breast cancer tissue was done by Biochain Institute Inc. Inflammatory breast cancer tissue sample (breast tumor tissue array T22350862-2) was also obtained from Biochain as well. Permission has been obtained according to the Declaration of Helsinki and following the specific authorization of the local Institutional Review Board (IRB) Committee to which the Chicago Medical School, Rosalind Franklin University of Medicine and Science refers (Institutional Review Board; IRB protocol 383 MIC). Since the tissue sections were commercially obtained from the BioChain Institute, Inc company, each sample is anonymous and blinded for laboratory research use. IHC was performed using primary antibodies against human OPG or IgG control as described previously [78]. Counterstaining was done by hematoxylin [78].

### OPG ELISA

The conditioned media of adherent HMEC, SUM149PT and SUM1315MO2 cells were collected, centrifuged and OPG levels were measured in the supernatants were measured by ELISA (Raybiotech) according to the manufacturer’s instructions. Results are expressed as the amount of OPG secreted (pg/ml) per 10^6^ cells.

### Proliferation assay

The Proliferating Index of cells with metabolically active mitochondria was determined by the 3-(4, 5-dimethylthiazol-2-yl)-2,5-diphenyl tetrazolium bromide (MTT)–based colorimetric assay (ATCC) as described previously [78]. Briefly, 4 × 10^4^ HMEC cells were allowed to grow into spheres in the presence of HMEC complete growth medium, conditioned media from SUM149PT and SUM1315MO2, OPG depleted conditioned media of SUM149PT and SUM1315MO2 and HMEC media reconstituted with 500 pg/ml or 1100 pg/ml recombinant human OPG in 24 well ultra-low attachment plates for 8 days. 100 μl of MTT reagent was added to all the sphere cultures after 8 days and further incubated for 4 h for the development of insoluble purple precipitate. Purple precipitate was solubilized in detergent and then read at 562 nm. The amount of MTT (yellow tetrazolium salt) that is converted to insoluble purple formazan crystals represents the number of proliferating cells.

### Cell cycle analysis by flow cytometry

HMEC spheres were grown in various media as previously described for cell proliferation assay [78]. Harvested spheres were trypsinized, cells were diluted to 10^6^ cells/ml and DNA distribution analysis was performed. Cells were fixed with 70 % methanol overnight and DNA was stained with propidium iodide (PI) at a final concentration of 50 mg/ml with RNaseA (100 U/ml) prior to flow cytometry analysis using LSRII (BD Biosciences). Results were analyzed using ModFit Lt V3 software (Verity Software House).

#### Stem cell analysis by flow cytometry

HMEC spheres were grown in various media as previously described for cell proliferation assay [78]. Harvested spheres were trypsinized and cells were diluted to 10^6^ cells/ml. Combinations of fluorochrome-conjugated monoclonal antibodies obtained from BD Biosciences (San Diego, CA, USA) against human CD44 (FITC) and CD24 (Alexa 647) or their respective isotype controls were added to the cell suspension at concentrations recommended by the manufacturer and incubated at 4 °C in the dark for 30 to 40 min. The labeled cells were washed in the wash buffer and DAPI was added to gate the live cells during the flow cytometry analysis using LSRII (BD Biosciences). Results were analyzed using ModFit Lt V3 software (Verity Software House).

#### In vitro capillary tube formation assay

HMEC, SUM149PT and SUM1315MO2 adherent cell conditioned media, OPG depleted SUM149PT and SUM1315MO2 conditioned media, and HMEC media reconstituted with 500 pg/ml or 1100 pg/ml recombinant human OPG were used for an *in vitro* capillary tube formation assay as per manufacturer’s instructions (BD Biosciences). Briefly, 10^4^ HMVEC-d cells were plated on a matrigel coated 96-well plate with different media, incubated for 16 h in 5 % CO_2_ at 37 °C, and examined for capillary tube formation under an inverted microscope and photographed. The assay was done in duplicate and each experiment was repeated three times.

### Western blot analysis

Cell or sphere protein lysates were quantitated by BCA assay. Equal amounts of protein (40 μg/lane) were separated on SDS-PAGE, electrotransferred to 0.45-mm nitrocellulose membranes, blocked with 5 % BSA, probed with antibodies of interest, and visualized using an enhanced-chemiluminescence (ECL) detection system.

### Cytokine profiling

Conditioned medium obtained from adherent and sphere cultures of HMEC, SUM149PT and SUM1315MO2 were spun at 1000 rpm for 10 min at 4 °C to remove the particulates and assayed for cytokine profiling using Raybiotech human cytokine antibody array AAH-CYT-7. The cytokine antibody array membranes were incubated with various conditioned media at 4 °C overnight. The membranes were washed, incubated with 1 ml of primary biotin-conjugated antibody at room temperature for 2 h, washed, incubated with 2 ml of horseradish peroxidase-conjugated streptavidin at room temperature for 45 min, and developed using ECL. Signal intensities were quantitated using an Alpha Inotech image analysis system. Signal intensities from all arrays were normalized to the same background levels with positive and negative controls using Raybiotech array AAH-CYT-7 analysis software.

### Human breast cancer qBiomarker copy number profiling

HMEC cells were allowed to make spheres in the presence of recombinant human OPG rich medium. HMEC spheres were cultured for three generations, each generation being of 7 days. Breast cancer spheres were also generated from SUM149PT and SUM1315MO2 cells and cultured for three generations. At the end of the third generation, DNA prepared from these spheres was used to profile qBiomarker Copy Number using the human Breast Cancer qBiomarker Copy Number PCR Array from SABiosciences. Apart from spheres, genomic DNA was also isolated from the inflammatory breast cancer patient tissue sample for profiling qBiomarker Copy Number. This array profiles the copy number of 23 genes reported to undergo frequent genomic alterations in human breast tumor DNA. Genes were chosen from the most frequently amplified or deleted genes relevant to oncogenic pathways and breast cancer biology based on the primary literature and public databases. The array analyzed each gene in each sample in quadruplicate and includes a stable multi-copy reference assay for accurate copy number determination via appropriate DNA input normalization. qBiomarker Copy Number PCR Arrays are the most reliable and sensitive copy number profiling technology for analyzing a panel of loci in signal transduction pathways or disease related gene networks.

## Additional files

Additional file 1: Figure S1. Depletion of OPG from breast cancer conditioned media. (PPT 162 kb)
Additional file 2: Table S1. Cytokine Profiling was done using Raybiotech array AAH-CYT-7 according to manufacturer's instructions. (PPT 173 kb)
Additional file 3: Table S2. Quantification of OPG staining in breast cancer tissue samples using Image J. (PPT 121 kb)
