# Supplementary figures and images for: Osteoprotegerin secreted by inflammatory and invasive breast cancer cells induces aneuploidy, cell proliferation and angiogenesis
Source: BMC Cancer. 2015 Nov 25;15:935. doi: 10.1186/s12885-015-1837-1 (PMC4660791; doi:10.1186/s12885-015-1837-1)

## Slide 1
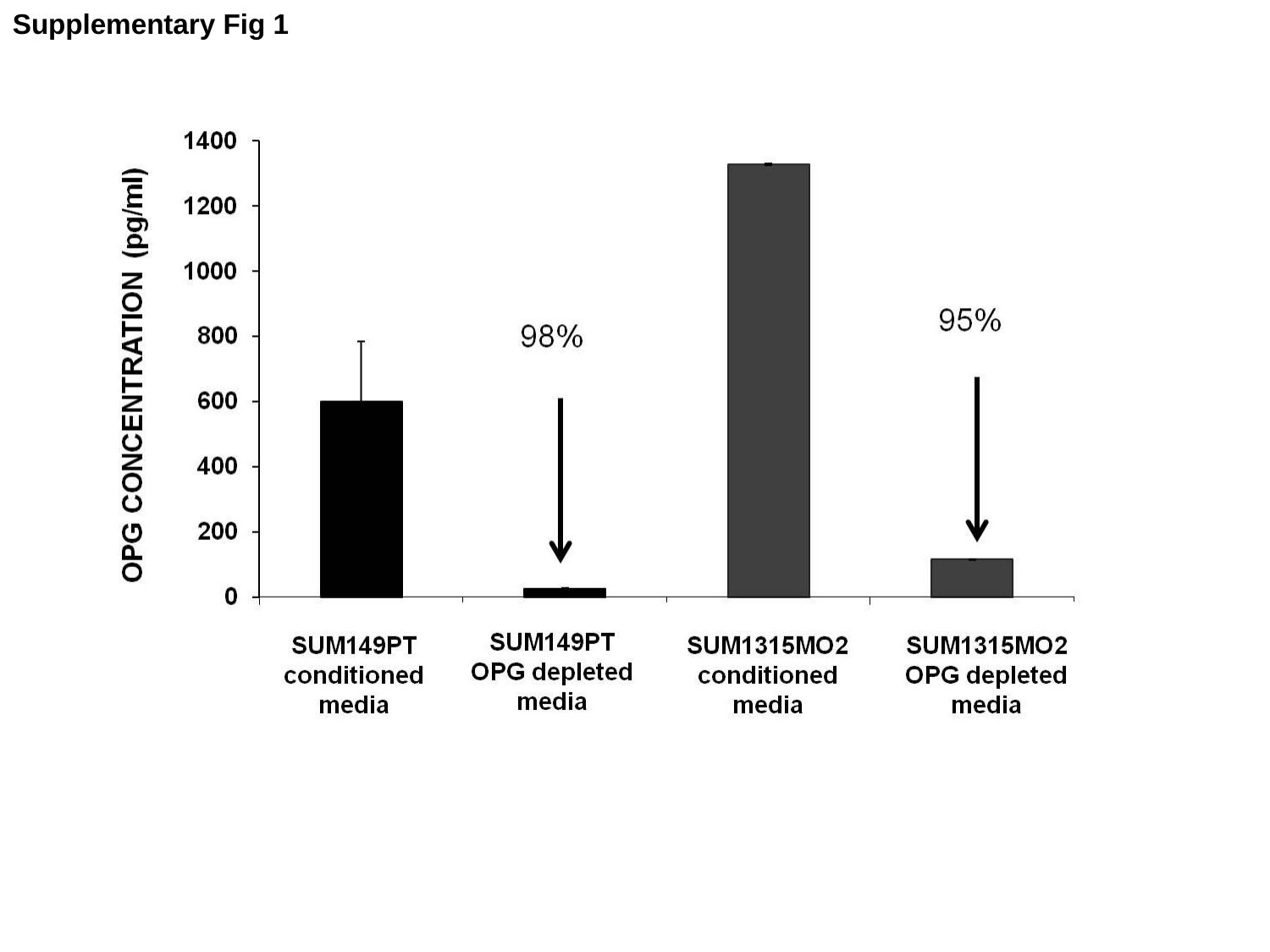

Supplementary Fig 1

Supplement: Additional file 1: Figure S1. — Depletion of OPG from breast cancer conditioned media. (PPT 162 kb) [file 12885_2015_1837_MOESM1_ESM.ppt]

## Slide 1
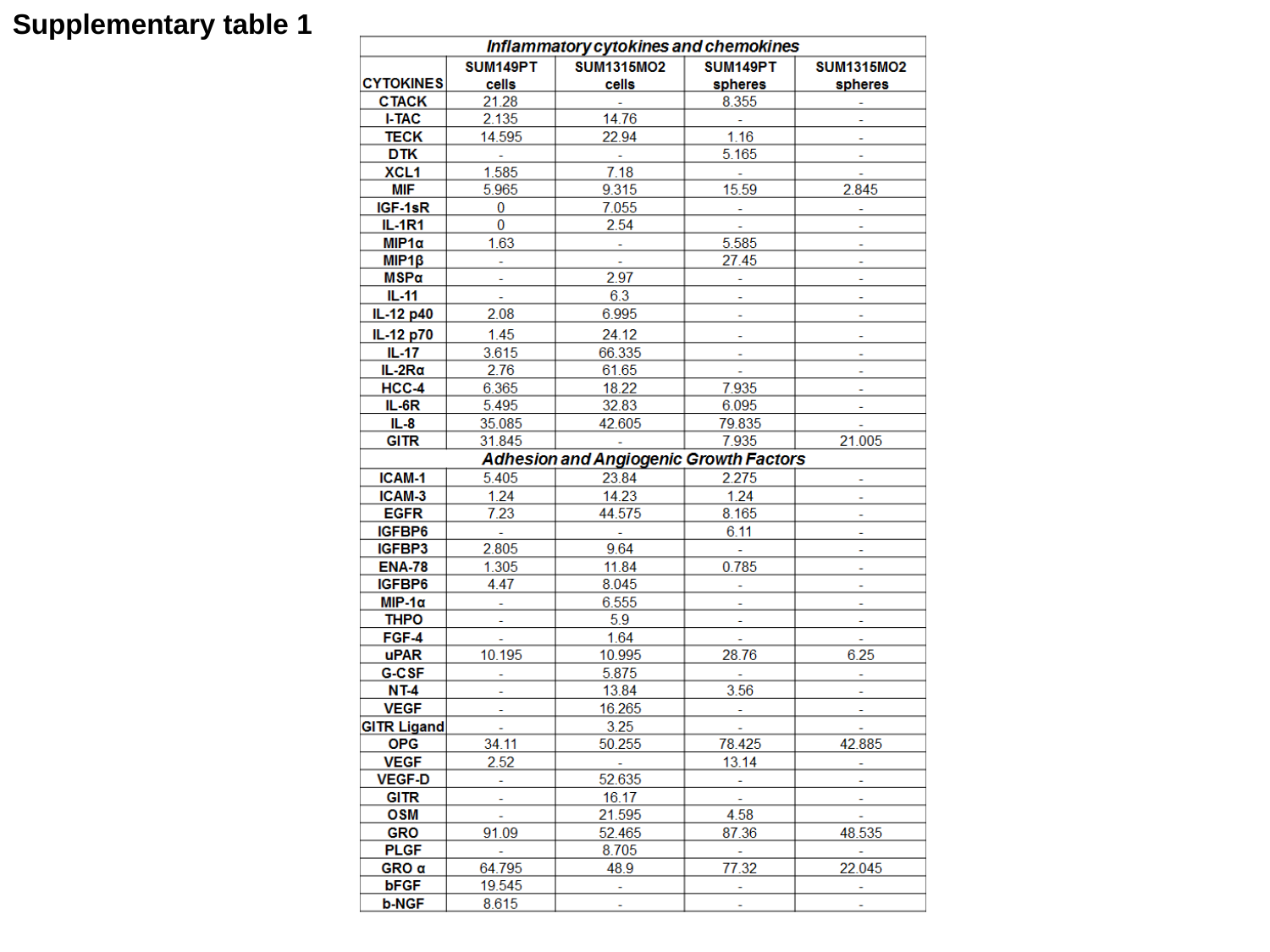

Supplementary table 1

Supplement: Additional file 2: Table S1. — Cytokine Profiling was done using Raybiotech array AAH-CYT-7 according to manufacturer's instructions. (PPT 173 kb) [file 12885_2015_1837_MOESM2_ESM.ppt]
