# Supplementary material for: Osteoprotegerin secreted by inflammatory and invasive breast cancer cells induces aneuploidy, cell proliferation and angiogenesis
Source: BMC Cancer. 2015 Nov 25;15:935. doi: 10.1186/s12885-015-1837-1 (PMC4660791; doi:10.1186/s12885-015-1837-1)
Supplement: Additional file 3: Table S2. — Quantification of OPG staining in breast cancer tissue samples using Image J. (PPT 121 kb) [file 12885_2015_1837_MOESM3_ESM.ppt]

## Slide 1
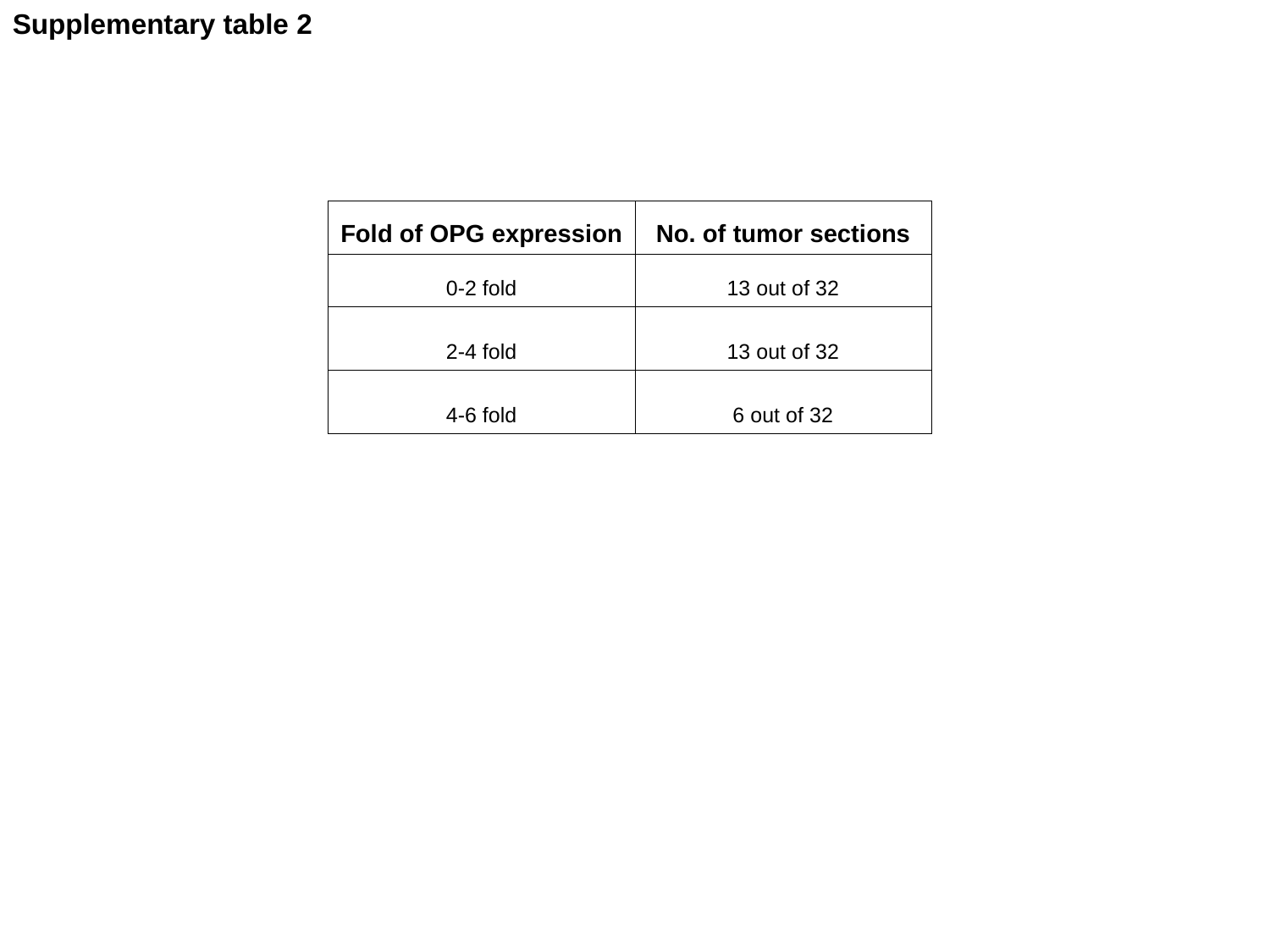

Supplementary table 2
| Fold of OPG expression | No. of tumor sections |
| --- | --- |
| 0-2 fold | 13 out of 32 |
| 2-4 fold | 13 out of 32 |
| 4-6 fold | 6 out of 32 |
